# A Novel Mechanism of 17-AAG Therapeutic Efficacy on HSP90 Inhibition in MYCN-Amplified Neuroblastoma Cells

**DOI:** 10.3389/fonc.2020.624560

**Published:** 2021-01-25

**Authors:** Reine Hanna, Jad Abdallah, Tamara Abou-Antoun

**Affiliations:** ^1^ Faculty of Sciences, Lebanese University, Fanar, Lebanon; ^2^ School of Pharmacy, Lebanese American University, Byblos, Lebanon

**Keywords:** neuroblastoma, HSP90, 17-AAG, IMR-32, SK-N-SH

## Abstract

**Background:**

Neuroblastoma is the most common pediatric extra-cranial nervous system tumor, originating from neural crest elements and giving rise to tumors in the adrenal medulla and sympathetic chain ganglia. Amplification of MYCN confers increased malignancy and poorer prognosis in high-risk neuroblastoma. Our SILAC proteomics analysis revealed over-expression of HSP90 in MYCN-amplified IMR-32 compared to the non-MYCN amplified SK-N-SH human neuroblastoma cells, rendering them highly resistant to therapeutic intervention.

**Methods:**

We used cellular bio-functional (proliferation, migration/invasion, apoptosis, viability and stem-cell self-renewal) assays and Western blot analysis to elucidate the therapeutic efficacy of HSP90 inhibition with 17-AAG.

**Results:**

17-AAG treatment significantly inhibited cellular proliferation, viability and migration/invasion and increased apoptosis in both cell lines. Moreover, drug treatment significantly abrogated stem-cell self-renewal potential in the MYCN-amplified IMR-32 cells. Differential tumorigenic protein expression revealed a novel mechanism of therapeutic efficacy after 17-AAG treatment with a significant downregulation of HMGA1, FABP5, Oct4, MYCN, prohibitin and p-L1CAM in SK-N-SH cells. However, we observed a significant up-regulation of p-L1CAM, MYCN and prohibitin, and significant down-regulation of Oct4, FABP5, HMGA1, p-ERK, cleaved/total caspase-3 and PARP1 in IMR-32 cells.

**Conclusions:**

HSP90 inhibition revealed a novel therapeutic mechanism of antitumor activity in MYCN-amplified neuroblastoma cells that may enhance therapeutic sensitivity.

## Introduction

Heat Shock Proteins (HSPs) have multiple roles in eukaryotic cells, however, they mainly serve as molecular chaperones having a central role in cell survival and development (1). They facilitate native protein folding and stabilization, protein translocation, protein re-folding and renaturation, or degradation for proteins that are beyond rescuing (2), hence ensuring protein quality control and preventing protein aggregation that would otherwise lead to apoptosis or necrosis (3). The role of HSPs is not only limited to normal conditions, rather they also become overexpressed when cells are exposed to any physiological or environmental stress, such as an increased temperature, ischemia, anoxia, toxins, ultra violet light, or viral particles (4).

HSPs are classified based on the molecular mass, where the 90-kDa group consists of two major isoforms: the inducible HSP90α and the constitutively expressed HSP90β (3). However, both HSP90α and HSP90β, referred to as HSP90, are studied as co-purified aggregates, due to the difficulty in their isolation (5).

The structure of HSP90 reveals three conserved domains: The N-Terminal Domain (NTD) containing the ATP binding site, the Middle Domain (MD) responsible for ATP hydrolysis and binding to client proteins, and the C-Terminal domain (CTD) destined for HSP90 dimerization (6). The NTD is linked to the MD by a charged linker region capable of modulating HSP90 activity (7). In addition, HSP90 contains a MEEVD motif (Met-Glu-Glu-Val-Asp) on the C-terminal end, mediating the interaction with co-chaperones containing tetratricopeptide repeat (TPR) domains (7).

Similar to other HSPs, HSP90 is a major molecular chaperone involved in the folding, stabilization, activation, and assembly of many proteins implicated in a wide range of biological processes under both normal and stress conditions, including several kinases, transcription factors, and signaling molecules (8, 9). The domains of HSP90 allow this chaperone to interact and affect its client proteins in three different ways: inducing the formation of the protein active conformation, or participating in the assembly of multiprotein complexes, or stabilizing the proteins to allow ligand binding and thus their activation (10). Moreover, HSP90 is also involved in the evolution and maintenance of disease states, such as cancer, by acting as a folding capacitor for unstable genetic variants, oncoproteins, or overexpressed proteins (11). In fact, many HSP90 client proteins are cancer-promoting molecules playing key roles in cancer development and progression, such as the Focal Adhesion Kinase (FAK) involved in cellular adhesion, the Epidermal Growth Factor Receptor (EGFR) and Protein Kinase B (AKT) that are involved in cell growth and proliferation, the Hypoxia-Inducible Factor 1α (HIF1α) that plays a role in cell survival and invasion and telomerase, which is involved in inhibiting cell death by inducing immortality, just to name a few (12). In addition, oncogenic mutations within tumor cells induce cellular “stress”, which requires additional control of the proteostasis where HSP90 interferes to maintain the survival of cancer cells (13).

Consequently, HSP90 has become a target in anticancer therapy because of its important roles in cancer development, particularly the regulation of tumor growth, cell proliferation, adhesion, migration, invasion, metastasis, angiogenesis, and apoptosis (13). Hence, various HSP90 inhibitors have been tested clinically. Because of the many HSP90 client proteins, these inhibitors target multiple signaling pathways simultaneously, making this therapy more promising in terms of efficiency in killing cancer cells *via* the multimodality therapeutic effect (12).

Particularly, the antibiotic geldanamycin is the first identified HSP90 inhibitor with its ability to bind to the ATP-binding pocket of the NTD (Jackson, 2013). However, this inhibitor shows poor solubility, limited stability and high hepatotoxicity in studies conducted *in vivo* (14). This led to the production of alternative derivatives of geldanamycin, such as tanespimycin (17-AAG), the first geldanamycin derivative to be tested in clinical trials (14).

17-AAG reversibly binds to the N-terminal ATP binding pocket in a competitive manner, inducing a conformational change within HSP90 (15). This modification is followed by the ubiquitination of its client proteins, leading to their degradation by the proteasome (15). Clinically, 17-AAG shows evidence of successful therapeutic activity in multiple malignancies, mainly in phase I and II clinical trials conducted on cases of HER2+ driven breast cancer, HER2 being a client protein of HSP90 (14).

In our current investigation, we aim at exploring the therapeutic effect of 17-AAG on neuroblastoma (NB), one of the most commonly diagnosed malignancy in infants. NB is a pediatric neuroendocrine solid tumor that commonly occurs in early childhood. It constitutes approximately 10% of the pediatric cancer cases, which makes it the most common extra-cranial solid tumor in pediatric patients, and leads to nearly 15% of cancer related deaths in children (16). NB originates from neural crest elements, and develops mainly in the medullary region of the adrenal glands and the sympathetic ganglia (17). Particularly, the most malignant, aggressive and high-risk NB tumors have been shown to present MYCN amplification, found in 20% of the tumors (18). Despite all the recent advances, inefficient therapies are still a barrier to overcome the poor clinical outcomes resulting after the therapeutic interventions in high-risk NB (19). Therefore, developing novel targeted therapies constitutes an important issue especially in the cases of high-risk NB.

Several cell lines have been engineered to be able to study NB *in vitro*, particularly, IMR-32, a MYCN amplified neuroblastic (N-type) adherent cell-line, obtained in 1967 from a biopsy of an abdominal mass of a 13-month-old Caucasian boy diagnosed with NB with rare areas of organoid differentiation (20). Interestingly, we previously reported that HSP90 was highly upregulated in these IMR-32 NB cells compared to the non-MYCN amplified SK-N-SH NB cells (21), thereby contributing to the growing interest in targeting this chaperone protein in high-risk NB cases. Therefore, due to its multi-targeted effects, inhibiting HSP90 in the MYCN-amplified human IMR-32 cell line would cripple all of its critical, tumor-promoting functions, thereby yielding tangible therapeutic effects on these malignant cells. Our studies in the IMR-32 cells are compared to the therapeutic effects of 17-AAG on the non-MYCN amplified SK-N-SH NB cell line.

Hence, we propose to target HSP90 by chemical inhibition using 17-AAG, and consequently assess the cellular bio-functions, including proliferation, viability, migration, and apoptosis. In addition, since many tumorigenic proteins are found to be upregulated in IMR-32 cells compared to the SK-N-SH cells, we propose to study their interactions with HSP90, as well as their implication in cellular functions and signaling pathways. This was conducted by examining the expression of proteins known to be upregulated in NB cells, and to detect their differential expression profile, as well as the interactions and crosstalk between them. Finally, we propose to examine the stem cell potency after drug treatment as these cells constitute the most aggressive sub-population of cancer stem cells, capable of driving tumorigenic behavior. We were able to demonstrate a potent anti-tumor, anti-stem cell enrichment and pro-apoptotic therapeutic effect of 17-AAG in our NB cells. Furthermore, differential protein expression after drug treatment indicated an interesting cross-talk between HSP90 and other tumorigenic pathways.

## Material and Methods

### Culture of Human Cell Lines

The IMR-32 (MYCN-amplified) and SK-N-SH (non MYCN-amplified) human NB cell lines were purchased from the American Type Culture Collection (ATCC, Manassas, VA, USA). These cells were cultured in minimal essential Eagle’s medium (EMEM; cat. No. M2279; Sigma) supplemented with 1% of 2 mM L-glutamine (cat. No. 7513; Sigma, St. Louis, MO, USA), 1% penicillin streptomycin (cat. No. P4333, Sigma), 1% of 1 mM sodium pyruvate (cat. No. S8636; Sigma), and 10% of fetal bovine serum (cat. No. F9665; Sigma) in T25 flasks at 5% CO_2_ and 37°C. The medium was replenished every 48 h, and the cells were passaged every two to three days when reaching an 80% confluence of the flask. The cells were then washed with Dulbecco’s phosphate-buffered saline 1X (PBS; D8537; Sigma), collected by trypsinization (cat. No. T3924, Sigma), and transferred into a 15 mL falcon tube to be centrifuged at 1,500 rpm for 5 min. The old medium was discarded, and the cells were re-suspended in 10 mL of fresh media and transferred into a T75 flask. The cells harvested from T75 flasks were frozen in Corning^®^Cryotubes (Corning Inc., New York, NY, USA) using 90% fetal bovine serum (FBS) and 10% DMSO (D2650; Sigma) to a final volume of 1 mL. The cryotubes were frozen at −20°C for 2 h first, then at −80°C for 24 h to provide a gradual decrease in temperature. Afterwards, they were transferred to liquid nitrogen for long term storage.

### WST-1 Assay

A total of 1.5x10^3^ IMR-32 and 3x10^3^ SK-N-SH cells were cultured in each well of a 96-well plate in a final volume of 100 μL. The “control wells” only contained growth media, and the “vehicle wells” were supplemented with 0.01% pure ethanol (this volume being equal to the needed volume of the drug). The drug Tanespimycin or 17-AAG (cat. No. ab141433, abcam), dissolved in 99% pure ethanol, was added in final concentrations of 0.5 and 1 μM to the “drug wells”. The cells were incubated for 24, 48, 72, and 96 h, and treated with 10 μL of WST-1 reagent (cat. No. ab155902; Abcam) per well prior to absorbance reading. The absorbance was detected after 3 h using a Synergy HTX Multi-Mode Microplate Reader at 450 nm (BioTek, Winooski, VT, USA).

### Cell Viability Assay

A 2.5x10^4^ IMR-32 and SK-N-SH cells were plated in each well of a 12-well plate to a final volume of 1 mL/well. After an overnight incubation to allow cell attachment, the medium was replaced and cells were incubated for 48 and 72 h with complete media, vehicle or drug in a final concentration of 1 μM. After each time point, the cells were collected, and a volume of each condition was mixed with an equal volume of trypan blue (cat. No. T8154; Sigma) for a 1:1 (V/V) dilution. 10 μL of this mix were then placed on a Neubauer Improved cell counting chamber which was examined under an optical microscope. Afterwards, cells were counted in four corners of the hematocytometer and the average was calculated. The number of viable cells was calculated as per the following formula: Viable cells/mL = average of viable cell count per square x dilution factor (=2) x 10^4^.

Cells viability was also obtained by calculating the following ratio: %Cell viability = [total viable cells (unstained)/total cells (viable + dead)] x 100.

### Apoptosis Assay

Cells were seeded in a 12-well plate with a density of 0.5x10^5^ cells/well. After 24 h, the medium was collected and replaced by the drug at a 1 μM concentration in a final volume of 1 mL, with the presence of control and vehicle wells. At 72 h after adding the drug, the medium was removed and replaced with 500 μL of PBS supplemented with 5% FBS containing the reagent CellEvent™ Caspase-3/7 (C10423; ThermoFisher Scientific) at a final concentration of 2 μM. After 30 min of incubation, random fields were picked and images of the fluorescent cells were captured using a Zeiss Axio Observer Microscope. Fluorescent and total cells were counted, and the percentage of fluorescent cells was calculated.

### Cell Migration Assay

Cells were seeded in a 12-well plate with a density of 2x10^5^ cells/well. When the cells reached 80-90% confluence, the complete growth medium was replaced by serum-free media in order to starve the cells overnight. The following day, the scratch was performed in a straight line across the center of the well using a 100 μL pipette tip. The wells were washed twice to remove any detached cells, and replenished with complete growth media containing the drug. Images were acquired immediately after performing the scratch and 24 h later. Wound healing was analyzed using AxioVision LE Application by determining the area of the wound at 0 and 24 h, and the percentage of wound closure was calculated by the formula: % of wound closure = [(wound area at 0 h – wound area after 24 h)/wound area at 0 h] x 100.

### Protein Interactions Analysis

Protein interactions were studied using STRING database (https://string-db.org/). This database serves to highlight functional enrichments as well as protein associations imported from other databases of curated biological pathway knowledge such as Gene Ontology (GO) and Kyoto Encyclopedia of Genes and Genomes (KEGG), which were used in our study to define molecular functions as well as interactions between the proteins of interest. These interactions include direct (physical) and indirect (functional) associations.

The proteins’ names were entered into the database, and a diagram of interactions was generated along with tables containing functions of these proteins, some of which were chosen to be displayed in a color-coded manner in our diagrams.

### Protein Extraction

Cells were seeded in 6-well plates at a density of 0.3x10^6^ cell/well. After 24 h, two protocols were followed depending on the proteins to be detected. For the p-ERK and ERK detection, the cells were starved for another 24 h with either the vehicle or the drug at a concentration of 1 μM. Then, prior to the collection of the cells, EGF (at 50 ng/mL) and FBS (5%) were added to the wells for 5 min to stimulate the phosphorylation; negative control wells were kept without stimulation. For the detection of the rest of the proteins, the medium was collected and replaced with the drug for 24 and 48 h. The cells were collected by scraping for protein extraction. Afterwards, the cells were centrifuged at high speed for 30 s at 4°C. The medium was then discarded, and the pellet was washed twice with PBS. The cells were then mixed, judging by the size of the pellet, with 80-120 μL of 2X Laemmli Buffer (cat. No. S3401; Sigma) used for denaturation and loading of protein samples. This buffer contains 4% SDS, 20% glycerol, 10% 2-mercapthoethanol, 0.004% btomophenol blue and 1.125 M Tris HCl, pH approx. 6.8. The Laemmli buffer was supplemented with 1% of protease/phosphatase inhibitors cocktail. The samples were then vortexed to be mixed with the Laemmli buffer, and centrifuged at 12,000 rpm and 4°C for 20 min. Finally, the samples were heated at 97°C for 5 min.

### Western Blot

Between 20 and 40 µg of total protein was electrophoresed on a TGX stain-free fastcast 10% SDS-PAGE gel (cat. No. 161-0183; Bio-Rad) for 90 min at 120 mV. Proteins were then transferred onto a methanol-activated PVDF membrane (cat. No. 162-0177; Bio-Rad) for 90 min at 90 mV and 4°C. Afterwards, membranes were blocked for 30 min using a blocking buffer prepared with TBS-Tween (0.1%) and 3% BSA (A2153; Sigma). Target proteins were detected using primary antibodies for L1CAM (rabbit polyclonal ab123990; abcam), p-L1CAM (ab61009; Abcam), MycN (84406; Cell signaling technology), Oct4 (2840S; Cell signaling technology), FABP5 (39926S; Cell signaling technology), HMGA1 (7777S; Cell signaling technology), PHB (2426S; Cell signaling technology), p-ERK (sc-7383; Santa Cruz Biotechnology) and ERK1/2 (4695S; Cell signaling technology) (dilution of 1:1,000 in blocking buffer); Cleaved-caspase 3 (9661, Cell signaling technology) and total caspase 3 (9662, Cell signaling technology); Cleaved-Parp1 (5625, Cell signaling technology) and total Parp1 (9523, Cell signaling technology). The blots were incubated with the primary antibodies overnight at 4°C, washed three times, 5 min each in TBS-Tween (0.1%), and incubated with the secondary antibody for 1 h at room temperature using the goat anti-rabbit IgG HRP-conjugated (cat. No. 170-5046; Bio-Rad) at a dilution of 1:1,000. The blots were then washed three times, 5 min each with 1x TBS-Tween (0.1%), incubated in Clarity Western ECL substrate (cat. No. 170-5061; Bio-Rad) and quantified by densitometric analysis using Image Lab software from Bio-Rad Laboratories. Stain free blot normalization was used instead of a housekeeping gene as a loading control.

### Sphere-Formation Assay

The Growth factor-reduced Matrigel™ (Cat. no. 354230; BD Biosciences) was thawed on ice at 4°C overnight prior to its use. IMR-32 and SK-N-SH cells (2,000 cells/well) were suspended in Matrigel™/serum free media (1:1 dilution). The solution was plated gently around the rims of the wells of a 24-well plate (50 μL per well). The matrigel was then allowed to solidify for 45 min in the incubator at 37°C. Meanwhile, two types of media were prepared: first, each 45 mL of NeuroCult™ NS-A Basal Medium (Human) (cat. No. 05750, Stemcell technologies) was supplemented with 5 mL of NeuroCult™ NS-A Proliferation Supplement (Human) (cat. No. 05751, Stemcell technologies), 100 μL of 10 μg/mL rh EGF (cat. No. 78006, Stemcell technologies), 50 μL of 10 μg/mL rh bFGF (cat. No. 78003, Stemcell technologies) and 50 μL of 0.2% Heparin (cat. No. 07980, Stemcell technologies) and second, EMEM media containing 5% FBS. For the treatment with 17-AAG, both media were supplemented additionally with concentrations of the drug of the order of 0.5 and 1 μM. Afterwards, 500 μL/well of either type of the prepared media were added to the center of the wells accordingly, and was regularly changed every two to three days. At day 7 after plating, images of the spheres were taken in order to measure the spheres diameter *via* Zeiss software. In addition, the number of spheres in each well was counted and the Sphere Formation Efficiency (SFE) was calculated as per the formula: SFE = (number of spheres counted/number of seeded cells) x 100.

### Statistical Analysis

Experiments were conducted in duplicates or triplicates, and repeated three independent times. The means ± the standard error of the mean (SEM) of all three experiments were calculated and plotted. Using GraphPad Prism software, statistical analysis was performed using Two-way ANOVA (WST-1 assay), one-way ANOVA (Sphere-formation assay), or Student’s t-test (Cell viability, apoptosis, migration and western blot assays) in order to determine statistically significant differences between the various groups in the experiments. Statistical significance was set as a p-value of < 0.05.

## Results

### 17-AAG Treatment Reduces Cellular Proliferation and Induces Cell Death in IMR-32 and SK-N-SH Cells

To determine the bio-functional cellular effects of the 17-AAG-mediated chemical inhibition of our target protein, HSP90, we first examined the proliferation rates of the MYCN-amplified IMR-32 compared to the non-MYCN-amplified SK-N-SH human NB cell lines over 96 h following the treatment. We focused on two effective drug concentrations: 0.5 and 1 μM.

**Figure 1:**
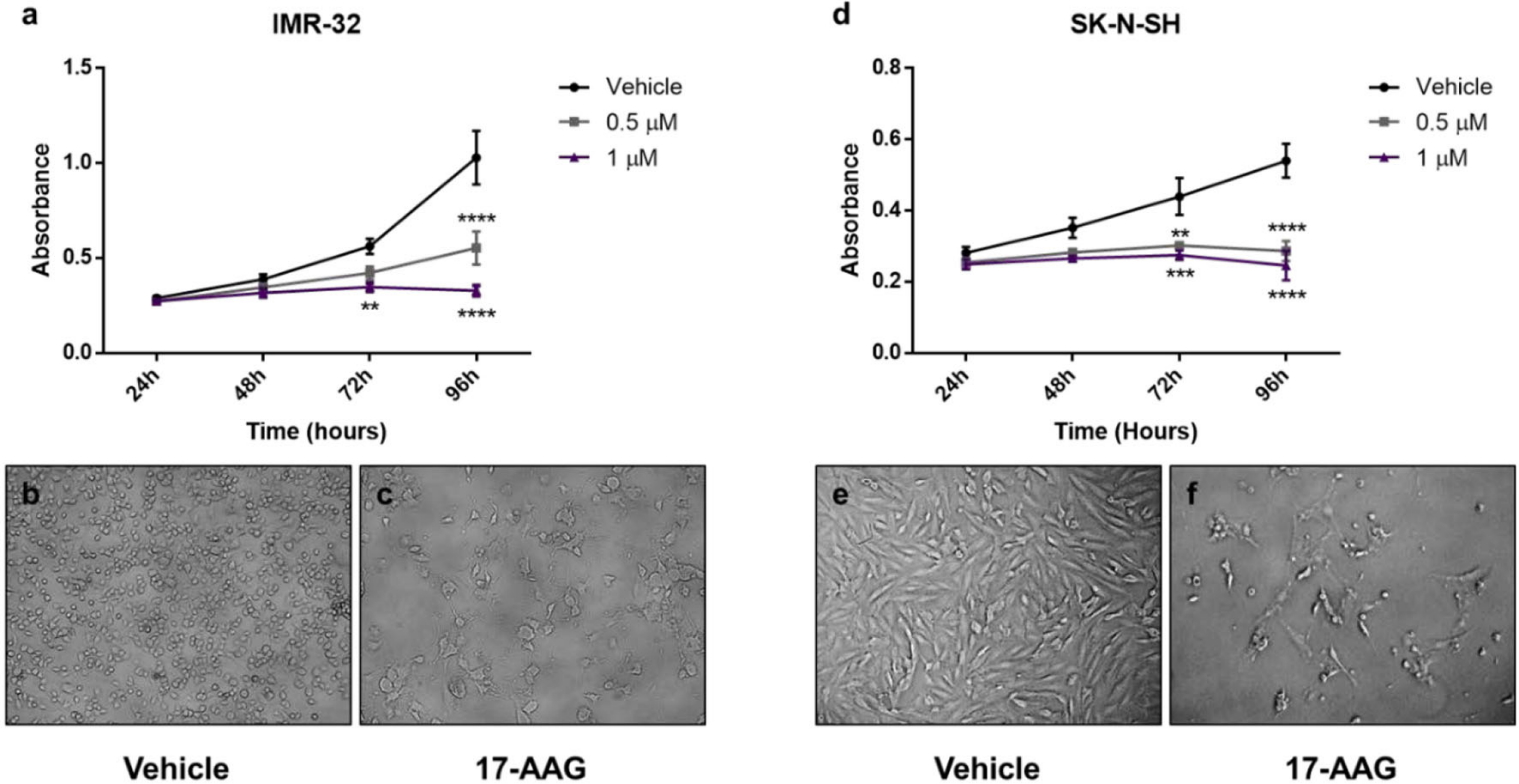
17-AAG treatment reduces the proliferation in IMR-32 and SK-N-SH cells. The WST-1 assay results show a decrease in the number of metabolically active cells after 17-AAG treatment with 0.5 and 1 μM concentrations in **(A)** IMR-32 and **(D)** SK-N-SH cells. Images of the wells at 96 h show a decreased number of IMR-32 and SK-N-SH cells, respectively, between **(B, E)** the vehicle and **(C, F)** the treated wells. The experiment was run in triplicate and repeated three times. Results represent the means ± standard error of the mean (SEM); **p < 0.01; ***p < 0.001; and ****p < 0.0001.

We found no significant difference in the proliferation rates between the vehicle and treated wells at 24 and 48 h post-treatment in both cell lines (**Figures 1A, D**).

However, in the case of IMR-32 cells, the absorbance values were significantly reduced (nearly by half) at 96 h post-treatment with a concentration of 0.5 μM (p<0.0001), while a more potent effect was observed with a concentration of 1 μM at 72 and 96 h (p<0.01 and p<0.0001, respectively) reflecting a lower number of metabolically active cells after the chemical inhibition of HSP90 (**Figure 1A**).

In parallel, the less aggressive cell line SK-N-SH, showed similar results but with a more prominent effect, especially at 72 h with 0.5 μM (p<0.01) and 1 μM (p<0.001) (**Figure 1D**).

This effect was supported by the visually distinguishable difference in viable cells observed in the vehicle-treated groups compared to the low number of cells visualized in the 1 μM drug-treated groups. In addition, a morphological difference was clear between the vehicle-treated and drug-treated cells, showing a more differentiated form with abnormal morphology in the drug-treated cells (**Figures 1B, C, E, F**).

Hence, a 1 μM concentration of 17-AAG was chosen to treat the cells in subsequent experiments due to its robust anti-proliferative effects on both our cell-lines at 72 and 96 h.

**Figure 2:**
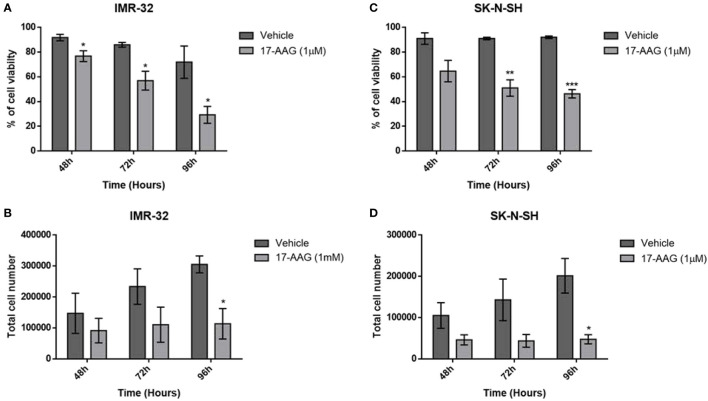
17-AAG treatment reduces cellular proliferation and viability in IMR-32 and SK-N-SH cells. The percentage of cell viability was significantly reduced at 72- and 96-hour post-treatment in comparison to the vehicle group in **(A)** IMR-32 and **(C)** SK-N-SH cells. Cell count showed a decrease in cellular proliferation after drug treatment, especially at 96 h in **(B)** IMR-32 and **(D)** SK-N-SH cells. The experiment was run in duplicate and repeated 3 times. Results represent the means ± SEM; *p < 0.05; **p < 0.01; and ***p < 0.001.

For further verification of the previous results, we explored the cell viability using the trypan blue exclusion assay. The results revealed a severe decrease in the percentage of cell viability of the IMR-32 cells in the 1 μM concentration group compared to the vehicle at 48, 72 and 96 h (p<0.05, **Figure 2A**). This decrease of cell viability was accompanied with a reduction of the total number of cells especially at 96 h (p<0.05, **Figure 2B**), and thus confirming the proliferation reduction observed in the WST-1 assay results.

Similar results were obtained with the SK-N-SH cell line, showing a severe decrease in cell viability especially at 72 and 96 h (p<0.001 and p<0.0001, **Figure 2C**), while the total number of cells was also significantly reduced, especially at 96 h (p<0.05, **Figure 2D**).

These results supported the values obtained with the WST-1 assay, showing that the decreased absorbance due to the treatment with 17-AAG could be explained by a decrease in proliferation and an increase in cell death.

### 17-AAG Treatment Induces Apoptosis in IMR-32 and SK-N-SH Cells

**Figure 3:**
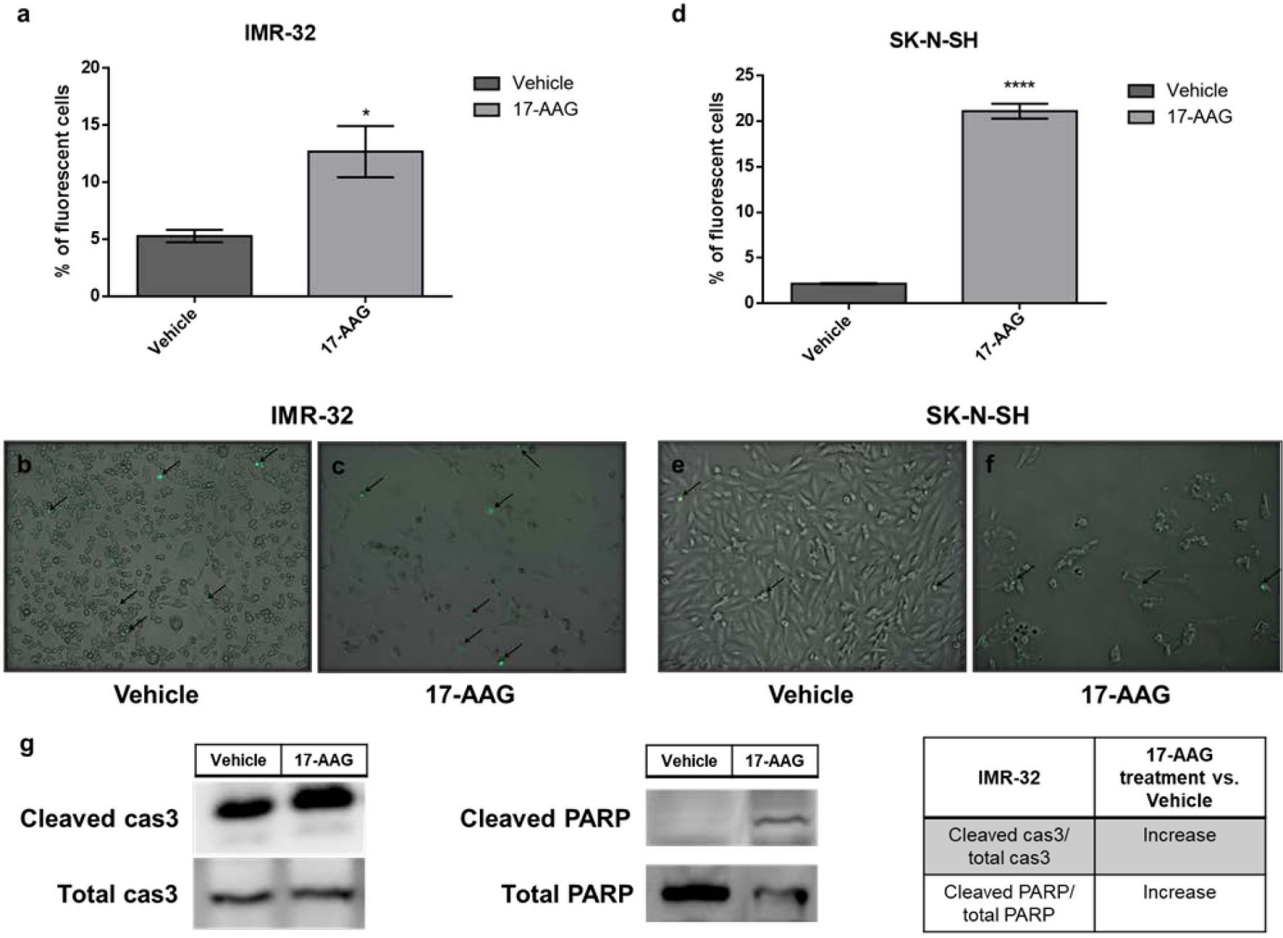
17-AAG treatment increases apoptotic rates in IMR-32 and SK-N-SH cells. The percentage of apoptotic fluorescent cells was significantly higher in **(A)** IMR-32 and **(D)** SK-N-SH cells treated with 17-AAG for 72 h. The green fluorescence represents apoptotic cells at a 10x magnification in **(B, E)** vehicle and **(C, F)** treated wells. **(G)** Expression of cleaved caspase 3 in comparison to total caspase 3 and cleaved PARP in comparison to total PARP. Black arrows indicate fluorescent apoptotic cells. The experiment was run in duplicate and repeated three times. Results represent the means ± SEM; *p < 0.05; and ****p < 0.0001.

In order to determine whether the increase in cellular death, seen in the previous results, was due to the induction of apoptosis by 17-AAG, we used the CellEvent Caspase3/7 kit capable of revealing the activation of these two mediators of apoptosis. In fact, a 1 μM concentration of 17-AAG treatment led to a significantly higher percentage of apoptotic cells represented by an increase in the percentage of fluorescent cells due to the activation of Caspases 3 and 7 in both cell types, IMR-32 and SK-N-SH (p<0.05 and p<0.0001, respectively). The induction of apoptosis in the IMR-32 cells was further confirmed by the significant increase (nearly doubling) of the expression of cleaved-caspase 3 and PARP1 in comparison to the total expression of these proteins at 72 h of treatment (**Figure 3**).

### 17-AAG Treatment Slows Down the Migration of IMR-32 Cells

**Figure 4:**
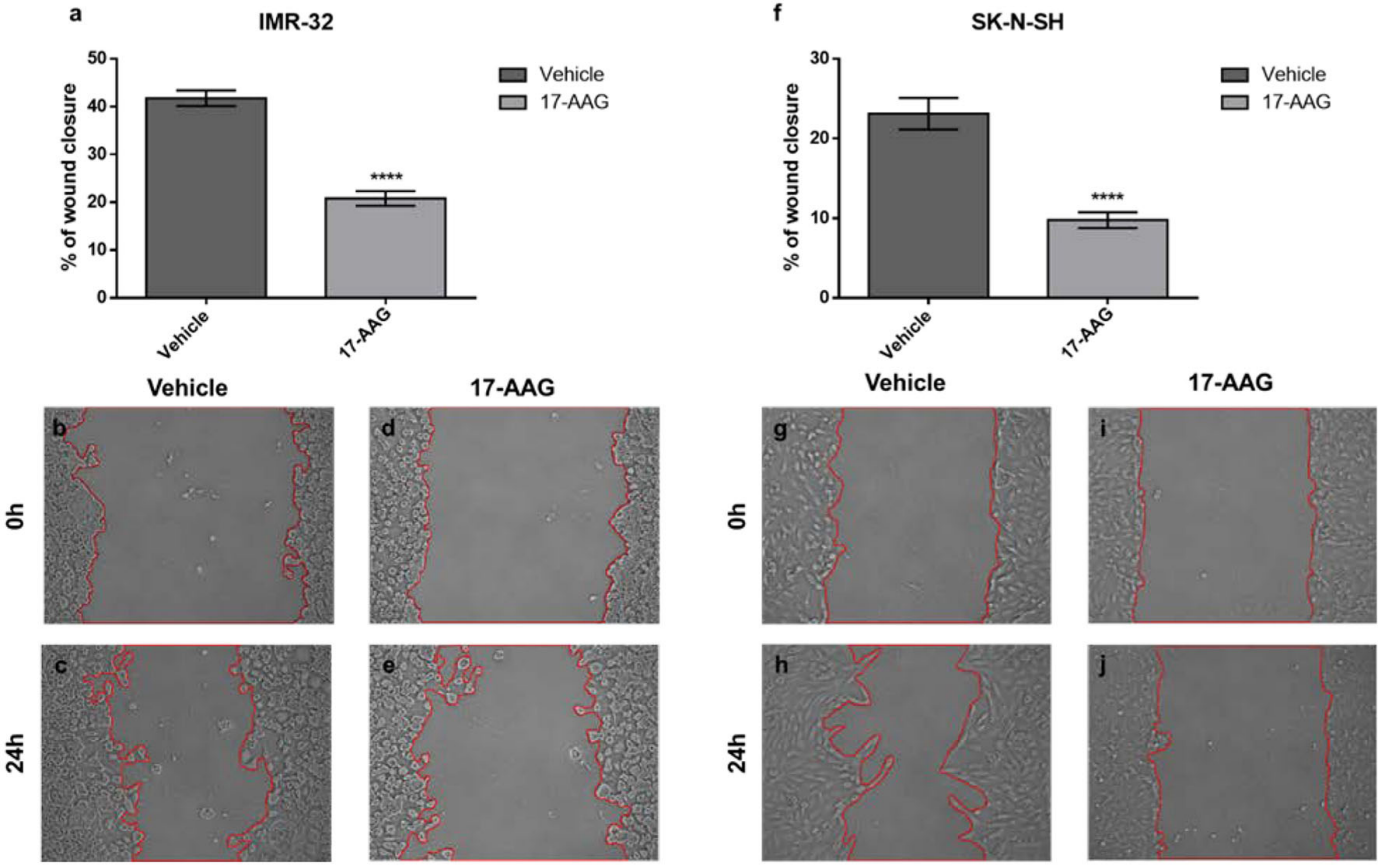
17-AAG treatment inhibits the migration of IMR-32 and SK-N-SH cells. The wound healing capacity significantly decreased after 17-AAG-treatment in **(A)** IMR-32 and **(F)** SK-N-SH cells. The 0-hour pictures were taken immediately after wound induction in **(B, G)** the vehicle and **(D, I)** 17-AAG groups. Twenty-four hours later, the pictures showed a 20–40% wound-closure in **(C, H)** the vehicle wells, whereas **(E, J)** the drug-treated groups showed less migration. The experiment was run in duplicate and repeated 3 times. Results represent the means ± SEM; ****p < 0.0001.

Next, we sought to examine the effect of the 17-AAG induced chemical inhibition of HSP90 on cellular migration, one of the cancer hallmarks. The wound healing migration assay revealed a reduced migratory capacity after treatment with 17-AAG as the wound closure area was decreased nearly by half post drug-treatment in IMR-32 (p<0.05) and SK-N-SH (p<0.0001) compared to vehicle-treated controls (**Figure 4**).

### Upregulated Proteins in IMR-32 Cells Exhibit an Important Interplay With the Tumorigenic HSP90 Protein

Next, we aimed to investigate the molecular mechanisms driving the effects on cellular bio-functions after 17-AAG treatment. To that end, we examined the protein expression of tumorigenic pathways before and after 17-AAG treatment to observe any alterations induced by the inhibition of HSP90. Initially, we analyzed the previously established interactions (21) between these tumorigenic proteins, which are overexpressed in IMR-32 in comparison to SK-N-SH cells, as well as their implications on cellular mechanisms and functions. For that purpose, we used STRING database to investigate the interplay between the following proteins: HSP90, L1CAM, HMGA1, PHB, Oct4 (POU5F1), MYCN, AKT, ERK (MAPK1), and FABP5.

**Figure 5:**
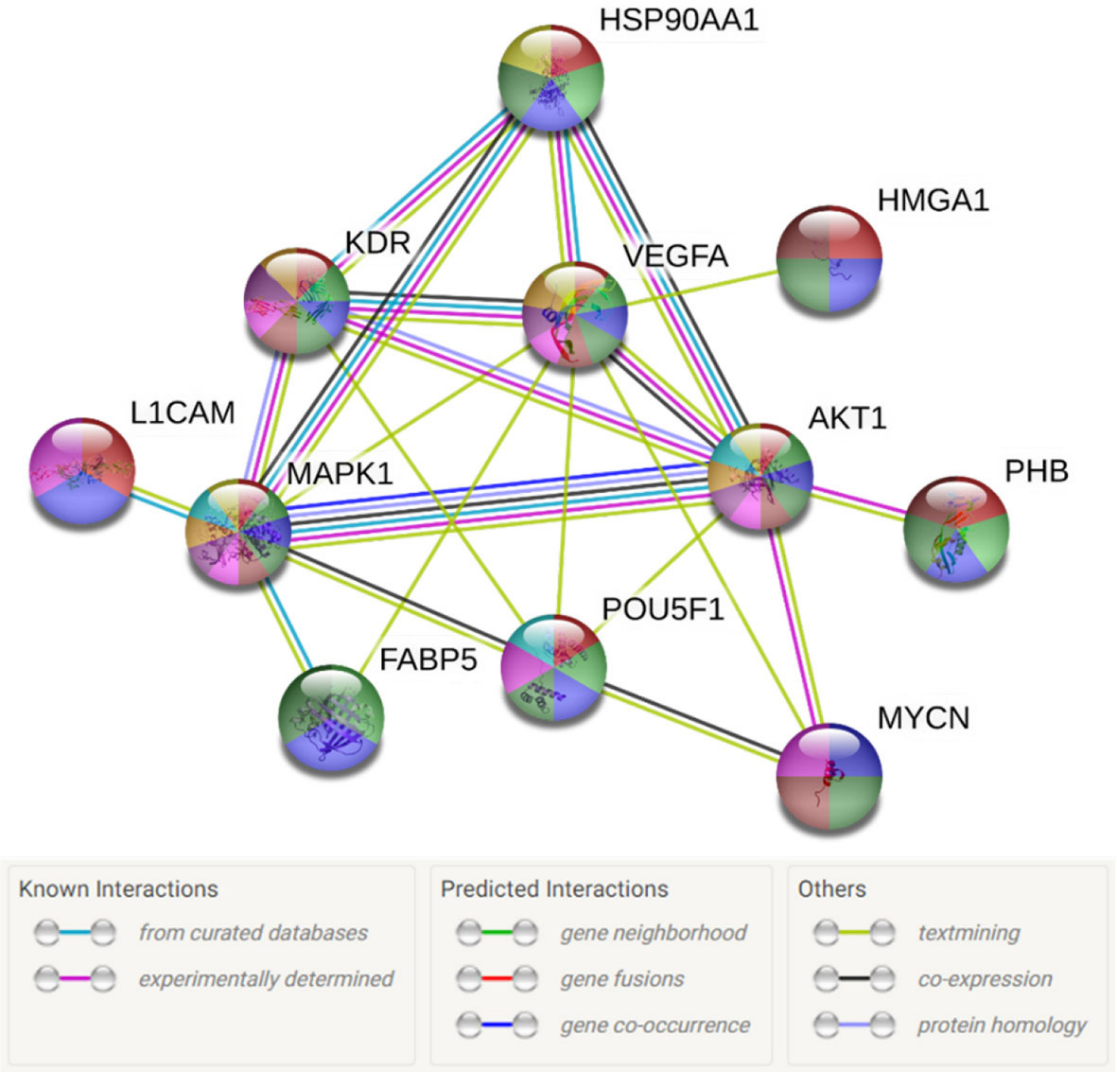
Diagram of the network of proteins interactions and their connections to HSP90. L1CAM, FABP5, PHB, MYCN, POU5F1, MAPK1 and HMGA1 were shown to be either directly or indirectly related to HSP90. The sources of these interactions are mainly, experimental results, curated databases and articles mentioning these molecules simultaneously. The node colors represent the molecular functions associated to each molecule figuring in **Tables 1** and **2**.

The interactions within this set of proteins highlighted a complex interplay between these molecules with the implication of VEGFR (KDR), known to be involved in angiogenesis, AKT, which plays a key role in cellular proliferation, metabolism, apoptosis and migration, and ERK (MAPK), which mediates biological functions such as cell growth, adhesion, survival and differentiation. Being directly or indirectly related to all these proteins, HSP90 constitutes a molecule contributing to diverse molecular pathways, especially pathways involved in cancer (**Figure 5**). In addition, according to data imported from functional classification systems such as GO and KEGG, these proteins contribute to various biological processes, molecular functions and pathways such as the regulation of cellular component organization, cellular processes, signal transduction, cell proliferation and differentiation, as well as VEGF and cancer signaling pathways (**Tables 1** and **2**).

**Table 1:** Biological processes and molecular functions of the proteins in the network in **Figure 5** imported from GO.

Pathway ID No.	Description	False discovery rate	Legend
GO:0051128	Regulation of cellular component organization	6.31e-05	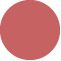
GO:0048522	Positive regulation of cellular processes	6.31e-05	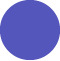
GO:0009967	Positive regulation of signal transduction	6.55e-05	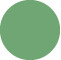
GO:0009889	Regulation of biosynthetic process	0.00021	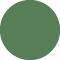
GO:0042127	Regulation of cell population proliferation	0.00029	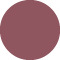
GO:0045595	Regulation of cell differentiation	0.00031	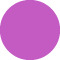

The colored circles or node colors in represent the molecular functions associated to each protein or molecule in **Figure 5**.

**Table 2:** KEGG pathways in which the proteins in the network in **Figure 5** participate.

Pathway ID No.	Description	False discovery rate	Legend
Hsa04370	VEGF signaling pathway	3.95e-06	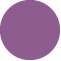
Hsa04510	Focal adhesion	8.08e-05	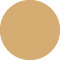
Hsa04550	Signaling pathways regulating pluripotency of stem cells	0.00040	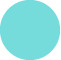
Hsa05200	Pathways in cancer	0.00087	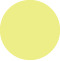

The colored circles or node colors in represent the molecular functions associated to each protein or molecule in **Figure 5**.

### 17-AAG Treatment Induces Differential Expression of Tumorigenic Proteins in IMR-32 in Comparison to SK-N-SH Cells

**Figure 6:**
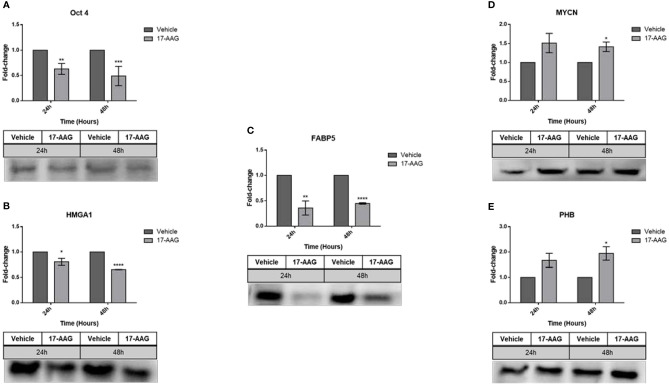
17-AAG treatment affects the expression of proteins known to be upregulated in IMR-32 cells. Following the chemical inhibition using 17-AAG in IMR-32 cells, western blot analysis revealed a significant down-regulation in the expression of **(A)** Oct4, **(B)** HMGA1, and **(C)** FABP5 at 24 and 48 h, as well as a significant increase in **(D)** MYCN and **(E)** PHB at 48 h following the 17-AAG treatment. The experiment was repeated three times. Normalization of the bands was conducted using the stain free blot method. Results represent the means ± SEM; *p < 0.05; **p < 0.01; ***p < 0.001; and ****p < 0.0001.

The interplay of these proteins in a direct or indirect manner to HSP90, triggered our interest to investigate the fluctuations in their expression after HSP90 inhibition. Western blot analysis of the IMR-32 total protein lysates (**Figure 6**) revealed a significant down-regulation of Oct4 (0.6 and 0.4 fold-change, respectively), HMGA1 (0.8 and 0.6 fold-change, respectively) and FABP5 (0.3 and 0.4 fold-change, respectively) after 24 and 48 h of 17-AAG treatment. However, 48 h post-treatment, MYCN (1.4 fold-change) and PHB (1.9 fold-change) were up-regulated following the inhibition of HSP90, in comparison to the vehicle-treated cells.

**Figure 7:**
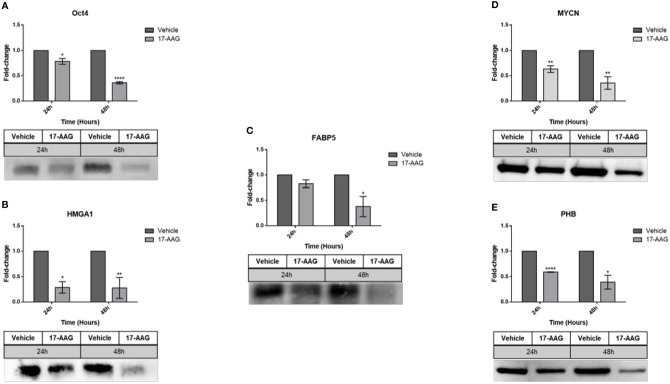
17-AAG treatment affects the expression of proteins in SK-N-SH cells. The treatment of SK-N-SH cells with 17-AAG led to the down-regulation of **(A)** Oct4, **(B)** HMGA1, **(C)** FABP5, **(D)** MYCN, and **(E)** PHB at 24 and 48 h. The experiment was repeated three times. Normalization of the bands was conducted using the stain free blot method. Results represent the means ± SEM; *p < 0.05; **p < 0.01; and ****p < 0.0001.

In parallel, this chemical inhibition in the SK-N-SH cell line (**Figure 7**) led to a significant decrease in the expression of all the aforementioned proteins at 24 and 48 h: Oct4 (0.7 and 0.3 fold-change, respectively), HMGA1 (0.3 fold-change), FABP5 (0.8 and 0.3 fold-change, respectively), MYCN (0.6 and 0.3 fold-change, respectively) and PHB (0.6 and 0.4 fold-change, respectively).

**Figure 8:**
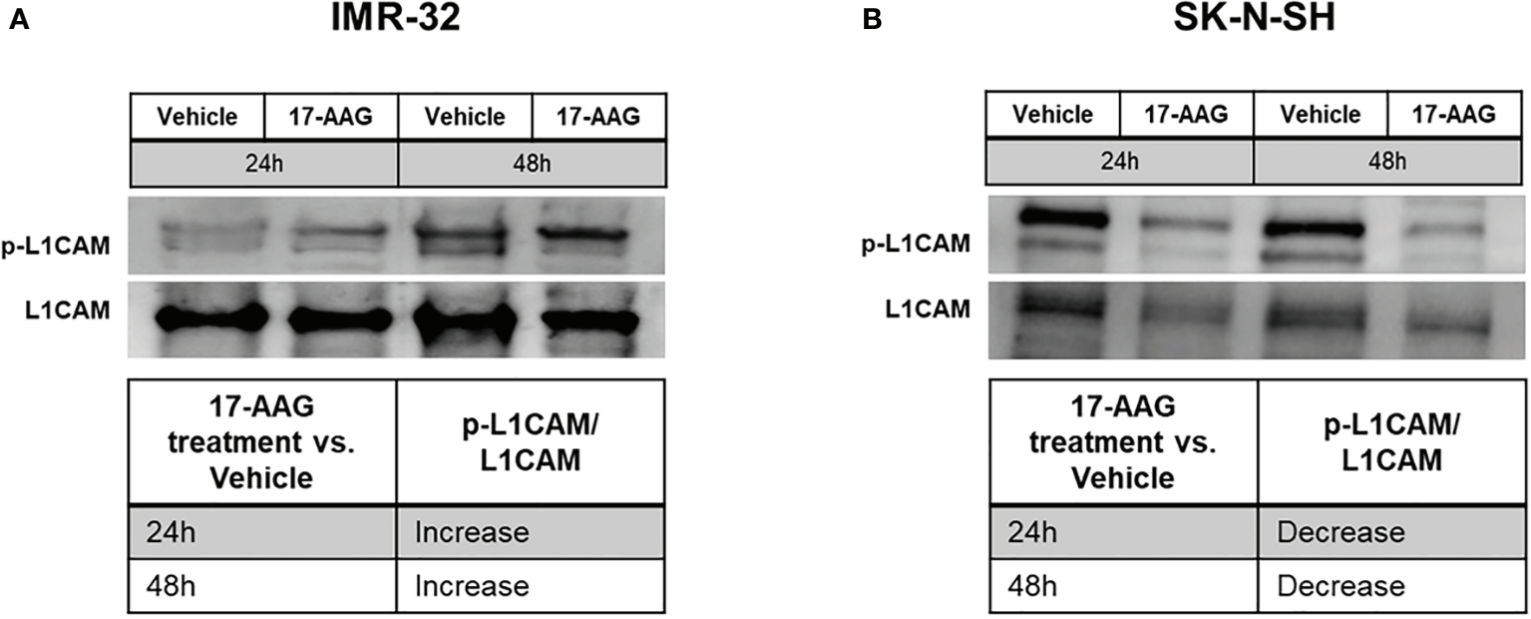
17-AAG treatment deregulated the phosphorylation of L1CAM in IMR-32 and SK-N-SH cells. The treatment of IMR-32 and SK-N-SH cells with 17-AAG led to **(A)** the increase of p-L1CAM/L1CAM levels in IMR-32 cells, and **(B)** decrease of the phosphorylation in SK-N-SH cells. The experiment was repeated three times. Normalization of the bands was conducted using the stain free blot method.

In addition, we analyzed the basal levels of phosphorylated L1CAM without any stimulation (with treatment in full serum media). By calculating the ratio between the levels of phosphorylated L1CAM to the levels of total L1CAM, the results showed an increase in the ratio of phosphorylation in the IMR-32 cells whereas SK-N-SH behaved in an opposite manner, showing a decrease in the levels of phosphorylated L1CAM (**Figure 8**).

**Figure 9:**
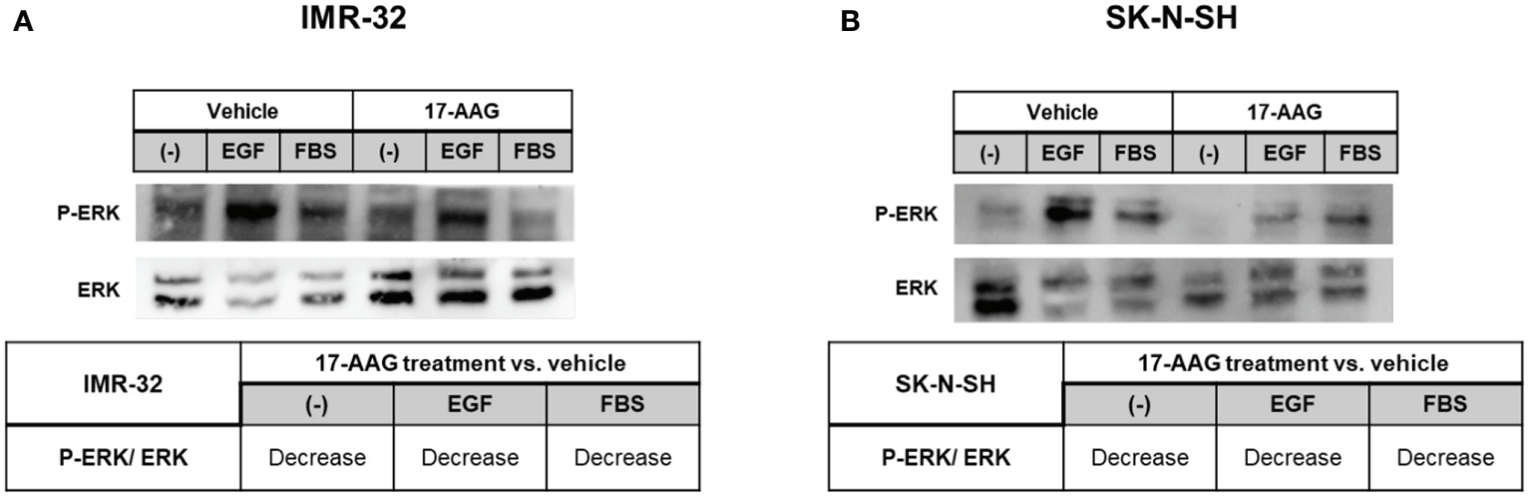
17-AAG treatment inhibited the phosphorylation of ERK in IMR-32 and SK-N-SH cells. 17-AAG treatment led to the decrease EGF- or FBS-induced ERK phosphorylation deduced by the reduction of p-ERK/ERK in **(A)** IMR-32 and **(B)** SK-N-SH cells. The experiment was repeated three times. Normalization of the bands was conducted using the stain free blot method.

Furthermore, we studied the phosphorylation of ERK, by starving the cells and treating them for 24 h, followed by stimulating ERK phosphorylation for 5 min with FBS or EGF. The analysis revealed a decrease in the ratio of phosphorylated ERK to total ERK, in all conditions and in both cell lines, showing that 17-AAG lowered the phosphorylation of this protein and thus its activation (**Figure 9**).

### 17-AAG Treatment Reduces the Stem Cell Capacity of Sphere Formation in IMR-32 Cells

**Figure 10:**
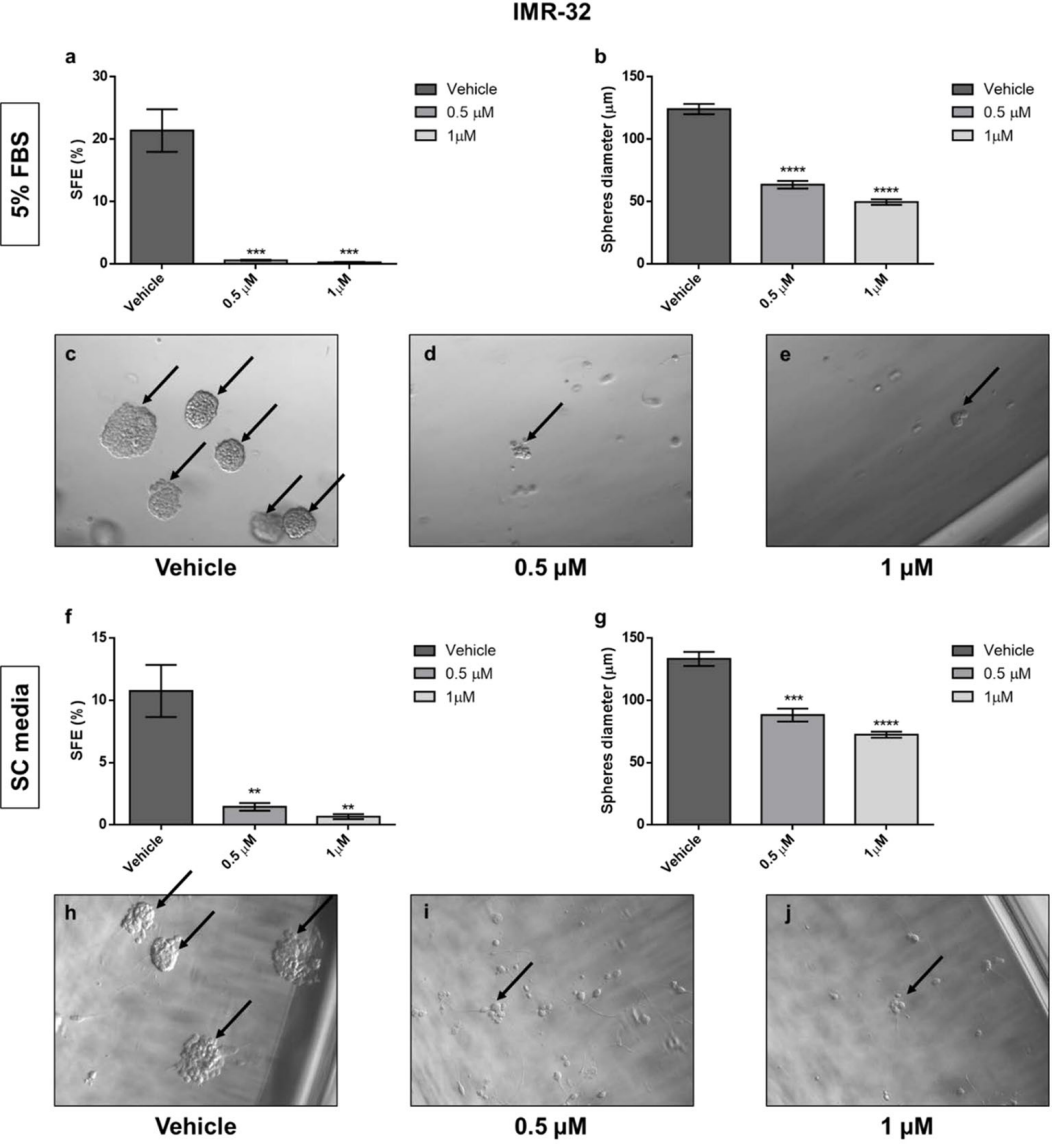
17-AAG inhibited the sphere-formation capacity of IMR-32 cells. The SFE determined by the ratio of the number of formed spheres to the number of seeded cells. The SFE was reduced in a highly significant manner after 17-AAG treatment with concentrations of 0.5 and 1 μM either using EMEM with **(A)** 5% media or **(F)** serum-free, stem cell media. **(B, G)** The spheres’ diameter, measured using Zeiss software, was significantly reduced due to the inhibition of HSP90. Black arrows indicate the spheres in **(C, H)** the vehicle, **(D, I)** 0.5 μM, and **(E, J)** 1 μM treated groups. The experiment was run in triplicate and repeated three times. Results represent the means ± SEM; **p<0.01; ***p<0.001; and ****p<0.0001.

The down-regulation of Oct4, one of the most known stem cell markers, after 17-AAG treatment in our cells, triggered our interest and led us to study the effect of the drug on the sphere formation of the cancer stem cells in our cell lines. Using two different types of media (EMEM containing 5% FBS and serum-free, stem cell media) gave us similar outcomes. The results showed an important reduction in the stem cell capacity of sphere formation in the IMR-32 cells (**Figure 10**) whereby SFE was severely reduced from 20% and 10% (5% FBS and SC media, respectively) in the vehicle-treated wells to less than 1% in the drug-treated wells with both 0.5 and 1 μM concentrations (p<0.01 and p<0.001). Moreover, the spheres’ diameter was reduced by nearly half after 17-AAG treatment, in comparison to the vehicle-treated wells (p<0.001 and p<0.0001). Yet, in the case of SK-N-SH cells, no spheres formed in either media conditions (data not shown) showing the lower levels of aggressiveness and invasiveness of this cell line due to the lack of stem-like sub-population of cells.

## Discussion

MYCN amplification in NB renders this devastating disease highly malignant and recurrence prone (21). Success rates in clinical trials are dismal despite the continued progress made towards devising targeted approaches to eradicate this cancer (22). Various reports have demonstrated the reason for treatment failure to be affiliated with the highly malignant, treatment-evasive and recurrence prone sub-population of cancer stem cells within the bulk tumor (23, 24). This sub-population of highly aggressive stem cells that makes up a small percentage of the bulk tumor, is able to recapitulate the original tumor and give rise to the heterogeneity of the bulk tumor mass (25). The heterogenic nature of the bulk tumor endows it with tumorigenic properties including potent proliferation rates, enhanced invasion and migration capacity, evasion of apoptosis, therapeutic resistance and cancer stem-cell maintenance and enrichment (25). We previously reported, using SILAC proteomics analysis, on the differential expression of tumorigenic proteins in NB IMR-32 and SK-N-SH cells and specifically the cancer stem-cell population residing within them (21, 26) before and after radio-therapy. HSP90 was found to be significantly up-regulated in the MYCN-amplified IMR-32 compared to the non-MYCN amplified SK-NS-H cells. Moreover, its expression was further significantly up-regulated in the sub-population of stem cells and after radio-therapy induction. This implicates the role played by HSP90 in the therapeutic response of these malignant cancer stem cells.

Our current study revealed significant anti-tumor activity after HSP90 inhibition with the drug 17-AAG in the malignant, MYCN-amplified IMR-32 cells and more potent anti-tumor activity in the non-MYCN amplified SK-N-SH cells. 17-AAG treatment significantly inhibited cellular proliferation and viability in both cell lines at 72 and 96 h post-treatment using as little as 0.5 and 1 µM dose of the drug. This inhibition was more potent in the non-MYCN amplified SK-N-SH cells compared to the MYCN-amplified IMR-32 cells indicating the added malignancy MYCN-amplification endows these cells with. Being a potent cancer proliferation driver (27), MYCN amplification contributes to the highly proliferative, malignant nature of the IMR-32 NB cells, which HSP90 inhibition with 17-AAG sufficiently abrogated. In addition, 17-AAG treatment significantly increased the rate of cellular apoptosis in both cell lines, but more potently in the non-MYCN amplified SK-N-SH cells. This further highlights the added resistance the MYCN-amplified IMR-32 cells possess in the face of therapeutic intervention. Resistance to apoptotic inducers is another hallmark of malignancy in cancers and the ability to reduce that resistance and increase cellular sensitivity to apoptotic compounds is of utmost importance.

Cancer metastasis in another major hallmark of malignancy and treatment failure. In order for cancer cells to metastasize they need to possess invasive and migratory potential (28, 29). Cancer stem cells are reported to acquire potent migratory and invasive characteristics (30) and we previously reported on the phenotypic reversible adaptive plasticity in NB cells under stressful conditions (31) that renders them highly migratory and treatment evasive. The ability of 17-AAG to significantly inhibit NB stem cell self-renewal and cellular migration in our model indicates a very important dual targeting and abrogation of both the migratory potential of the malignant cells as well as the self-renewal of the stem cell sub-population. Eliminating only the bulk tumor cells while leaving behind the highly aggressive, malignant and recurrence-prone cancer stem cells only buys us time before these cells recapitulate the original tumor leading to recurrence of treatment evasive cancers. It is of utmost importance that therapeutic interventions effectively eliminate both the bulk tumor cells as well as the sub-population of cancer stem cells to completely eradicate the cancer. 17-AAG exhibited potent inhibition on the cancer stem cell self-renewal potential as well as anti-proliferative, anti-migratory and pro-apoptotic activity on the bulk NB tumor cells in our model. As such, HSP90 inhibition effectively eliminated the bulk NB cancer cells and also eliminated the cancer stem cell sub-population, thereby providing very promising potential for this therapeutic intervention to completely eradicate the cancer, prevent metastatic spread and reduce the chance of malignant tumor recurrence.

In order to elucidate the molecular mechanisms behind this therapeutic effect of 17-AAG, we investigated the protein expression profile of various tumorigenic targets including Oct4 (enhances stem cell self-renewal) MYCN, FABP5 (enhances angiogenesis), HMGA1 (enhances cell migration), Prohibitin (PHB, enhances proliferation and suppresses differentiation) and L1CAM (enhances stem cell self-renewal), cleaved-caspase 3 and cleaved PARP1 (apoptotic inducers). While HSP90 inhibition led to the significant down-regulation of Oct4, HMGA1 and FABP5 at 24 and 48 h post-treatment in IMR-32 cells, it led to the significant up-regulation of MYCN and PHB 48 h post-treatment. The simultaneous down-regulation of these tumorigenic proteins sheds interesting and novel insight on the therapeutic mechanism of 17-AAG treatment. This explains the inhibition on cellular proliferation, migration, viability, stem-cell self-renewal and enhanced apoptosis. In fact, we confirm that the increased rate of 17-AAG induced apoptosis is dependent on cleaved-caspase 3 and PARP1 as both proteins were found to be significantly up-regulated 72 h post drug treatment in IMR-32 cells.

A possible explanation for the MYCN and PHB over-expression in IMR-32 cells after drug treatment could be that these malignant cells, who are highly dependent on the amplification of MYCN, may exhibit this compensatory up-regulation of these tumorigenic proteins in an attempt to evade this chemical inhibition of HSP90. This attempted compensatory mechanism of drug-evasion was ineffective in preventing the drug induced anti-tumor effects on these cells. In support of our findings, we previously reported on the compensatory up-regulation of L1CAM in IMR-32 cells post radio-therapy in an attempt to evade this conventional therapeutic intervention (26). In contrast, SK-N-SH cells treated with 17-AAG exhibited a significant down-regulation of all the aforementioned proteins including MYCN and PHB. These cells perhaps do not possess the molecular machinery to undergo the compensatory up-regulation of MYCN and PHB in the face of the potent HSP90 inhibition with 17-AAG.

More interestingly, we assessed the basal level of phosphorylated L1CAM, a stem cell maintenance marker, in both IMR-32 and SK-N-SH cells before and after HSP90 inhibition with 17-AAG. The basal level of phosphorylated L1CAM significantly increased in the IMR-32 cells, whereas it decreased in the SK-N-SH cells. This further implies that the malignant, MYCN-amplified IMR-32 cells possess a molecular machinery distinct from that in the less malignant, non-MYCN amplified SK-N-SH cells that endows them with the ability to differentially express tumorigenic proteins and activate them as a compensatory attempt in response to therapeutic intervention. Despite this increase in p-L1CAM after 17-AAg treatment in IMR-32 cells, their stem-cell self-renewal potential was significantly inhibited with 17-AAG treatment, possibly due to the significant down-regulation of the stem marker Oct-4.

In an attempt to delineate the mechanism of reduced migration capacity after 17-AAG treatment, we investigated the epidermal growth factor (EGF)- and fetal bovine serum (FBS)-induced phosphorylation of ERK1/2, a potent mitogenic factor and enhancer of cellular migration, in both IMR-32 and SK-N-SH cells 24 h after drug treatment. EGF- and FBS-induced phosphorylation of ERK1/2 was significantly reduced after 24 h of 1 µM 17-AAG treatment. To the best of our knowledge, we are the first to report on the inhibition of EGF-induced phosphorylation of EKR1/2 with 17-AAG treatment. While 17-AAG binds to the ATP-binding cassette of HSP90, it appears to also simultaneously inhibit EGF-induced phosphorylation of down-stream signaling cascades such as the mitogen activator ERK1/2.

## Conclusion

High risk NB with MYCN amplification typically presents with highly malignant disease that readily metastasizes and recurs after initial treatment, thereby leading to very poor prognosis. The heat shock chaperon protein HSP90 was found to be highly over-expressed in the MYCN-amplified IMR-32 NB stem cells, especially after radio-therapy. We showed a significant reduction in cellular proliferation, migration and viability, whereas enhanced apoptosis in the IMR-32 cells after HSP90 chemical inhibition with 17-AAG treatment. Moreover, the stem cell self-renewal potential of these cells was also significantly inhibited with 17-AAG treatment. These therapeutic effects were attributed to the differential expression of tumorigenic proteins after HSP90 inhibition. Our investigation is not without limitation. First we only examined the effects of 17-AAG on 2D and 3D cell culture models. In our future investigations, we aim to examine the therapeutic effects of 17-AAG in a murine model of tumorigenicity to determine the in-vivo therapeutic effects of the drug. In addition, previous reports have shown that HSP90 inhibition with the N-terminal inhibitor 17-AAG leads to compensatory up-regulation of HSP27, 40 and 70 (32), which may explain the lack of clinical efficacy using 17-AAG in some cancers. As such, our future studies include using a combinatorial therapy of HSP90 with HSP70 inhibition in order to combat this compensatory mechanism. Moreover, we further plan to conduct combinatorial therapy using 17-AAG treatment with MYCN and PHB targeted therapy to inhibit the proteins significantly up-regulated after 17-AAG treatment in our model. This will allow us to determine if a synergistic effect of dual versus single therapy exists. Perhaps combinatorial inhibition of the up-regulated targets after 17-AAG treatment would further cripple these malignant, MYCN-amplified cancer cells yielding more significant anti-tumor effects with the combination therapy.

## Data Availability Statement

The original contributions presented in the study are included in the article/**Supplementary Material**. Further inquiries can be directed to the corresponding authors.

## Author Contributions

Conceptualization, JA and TA-A. Methodology, JA, RH, and TA-A. Formal analysis, JA, RH, and TA-A. Investigation, JA, RH, and TA-A. Writing—original draft preparation, JA, RH, and TA-A. Writing—review and editing, JA, RH, and TA-A. Supervision, JA and TA-A. Project administration, JA and TA-A. Funding acquisition, JA and TA-A. All authors contributed to the article and approved the submitted version.

## Funding

This research was funded by the Lebanese National Council for Scientific Research, grant number CNRS-848, and The Lebanese American University (LAU), grant number SRC-2019-15.

## Conflict of Interest

The authors declare that the research was conducted in the absence of any commercial or financial relationships that could be construed as a potential conflict of interest.
