# Supplementary material for: A Novel Mechanism of 17-AAG Therapeutic Efficacy on HSP90 Inhibition in MYCN-Amplified Neuroblastoma Cells
Source: Front Oncol. 2021 Jan 25;10:624560. doi: 10.3389/fonc.2020.624560 (PMC7868539; doi:10.3389/fonc.2020.624560)
Supplement: Supplementary file 1 [file DataSheet_1.docx]

Supplementary Material

# Supplementary Figures and Tables

## Supplementary Figures


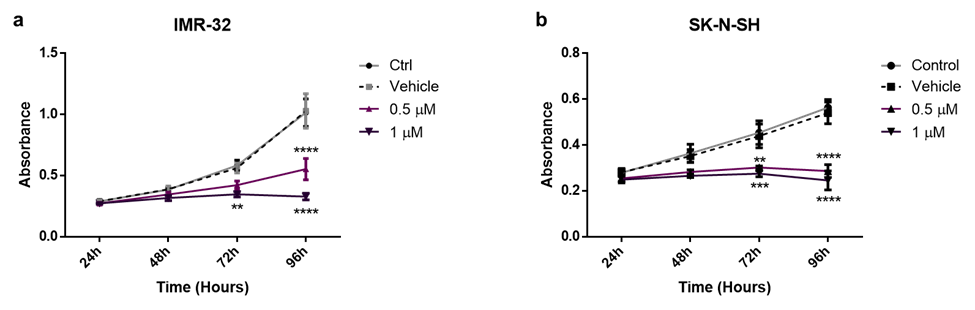


**Supplementary Figure 1.** The control (non-treated) and vehicle (treated with ethanol) showed no difference in the WST-1 assay.


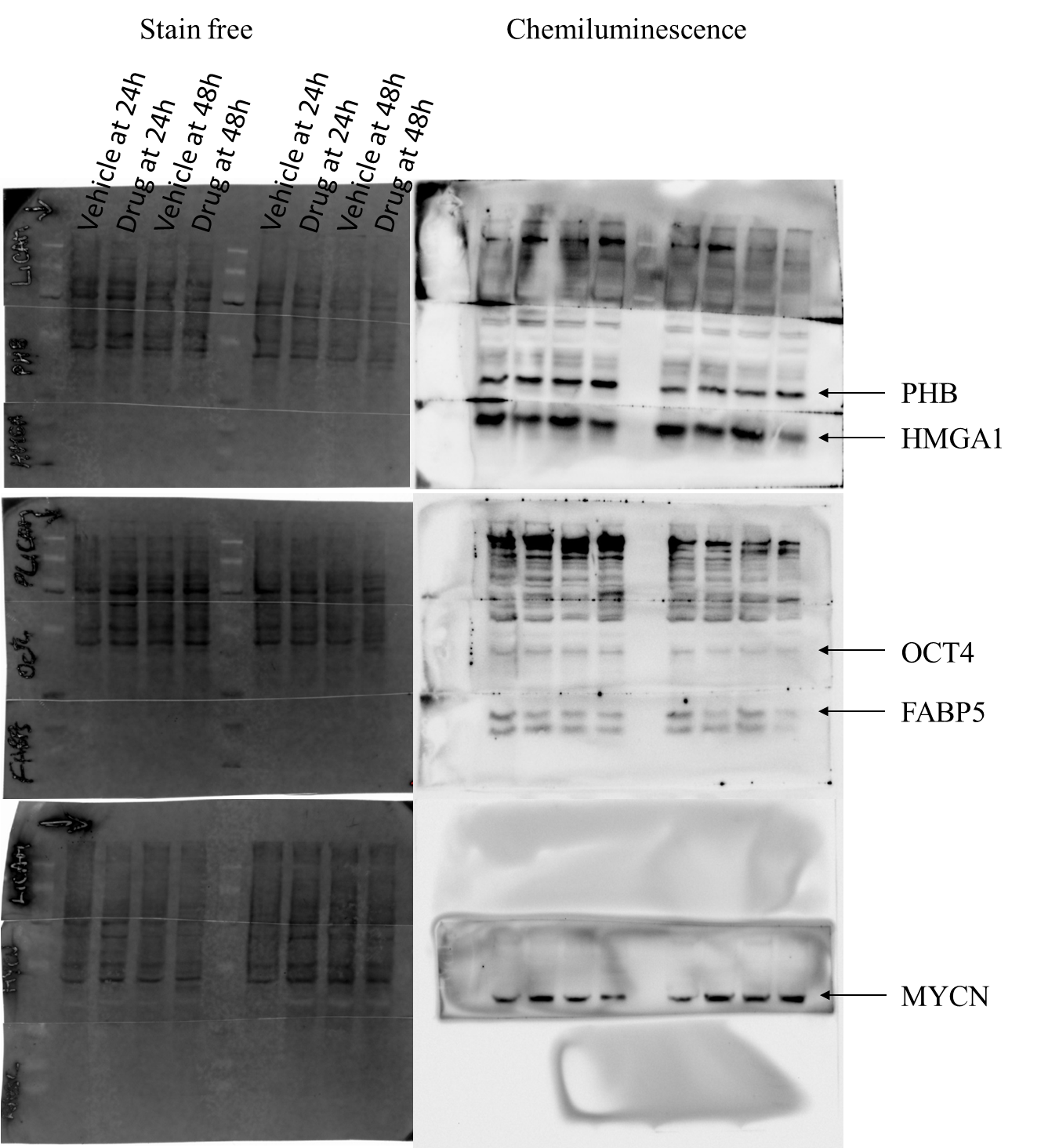


**Supplementary Figure 2.** Stain free blots and chemiluminescent images of the different proteins detected via western blotting in IMR-32 cells.


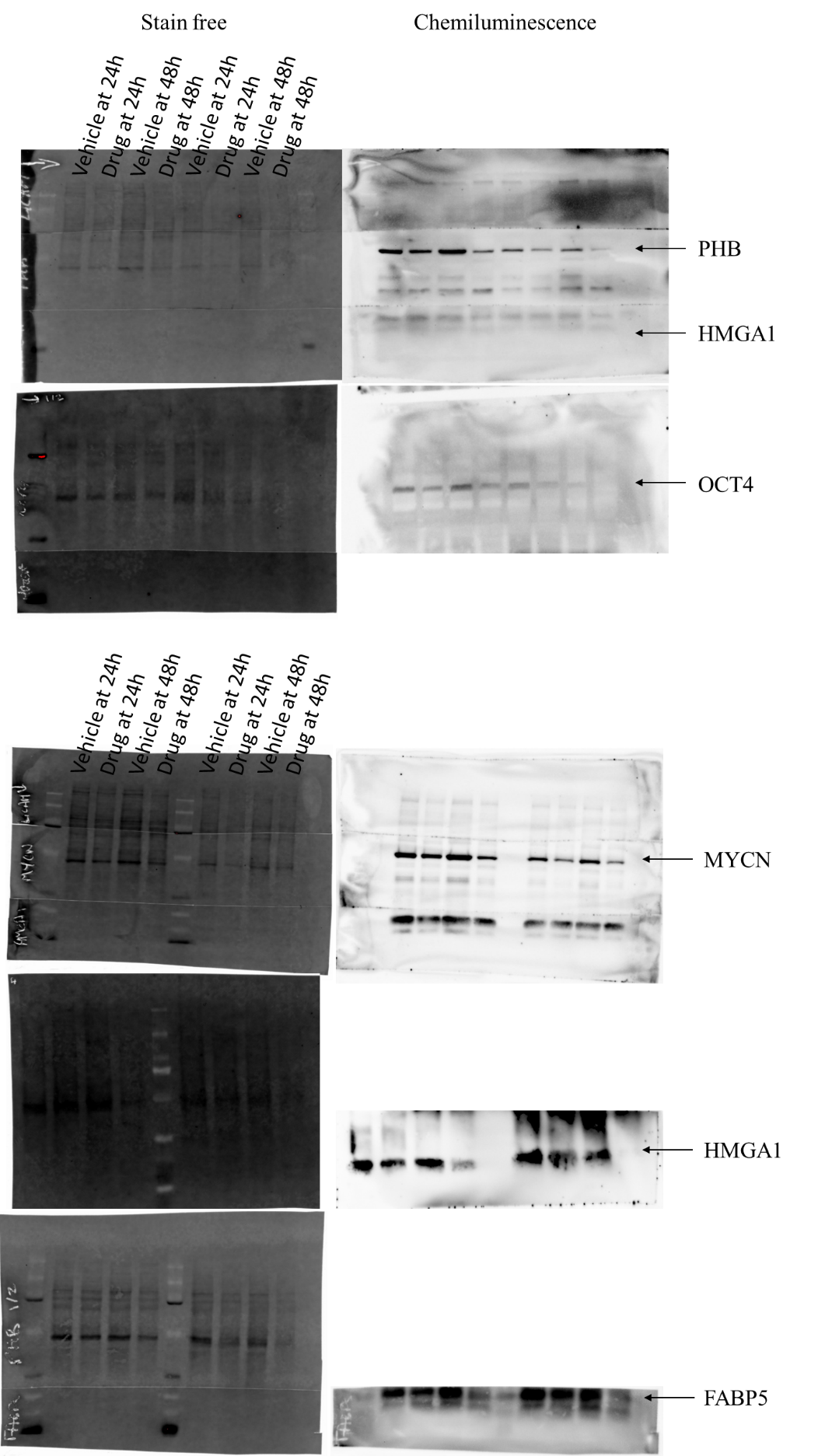


**Supplementary Figure 3.** Stain free blots and chemiluminescent images of the different proteins detected via western blotting in SK-N-SH cells.


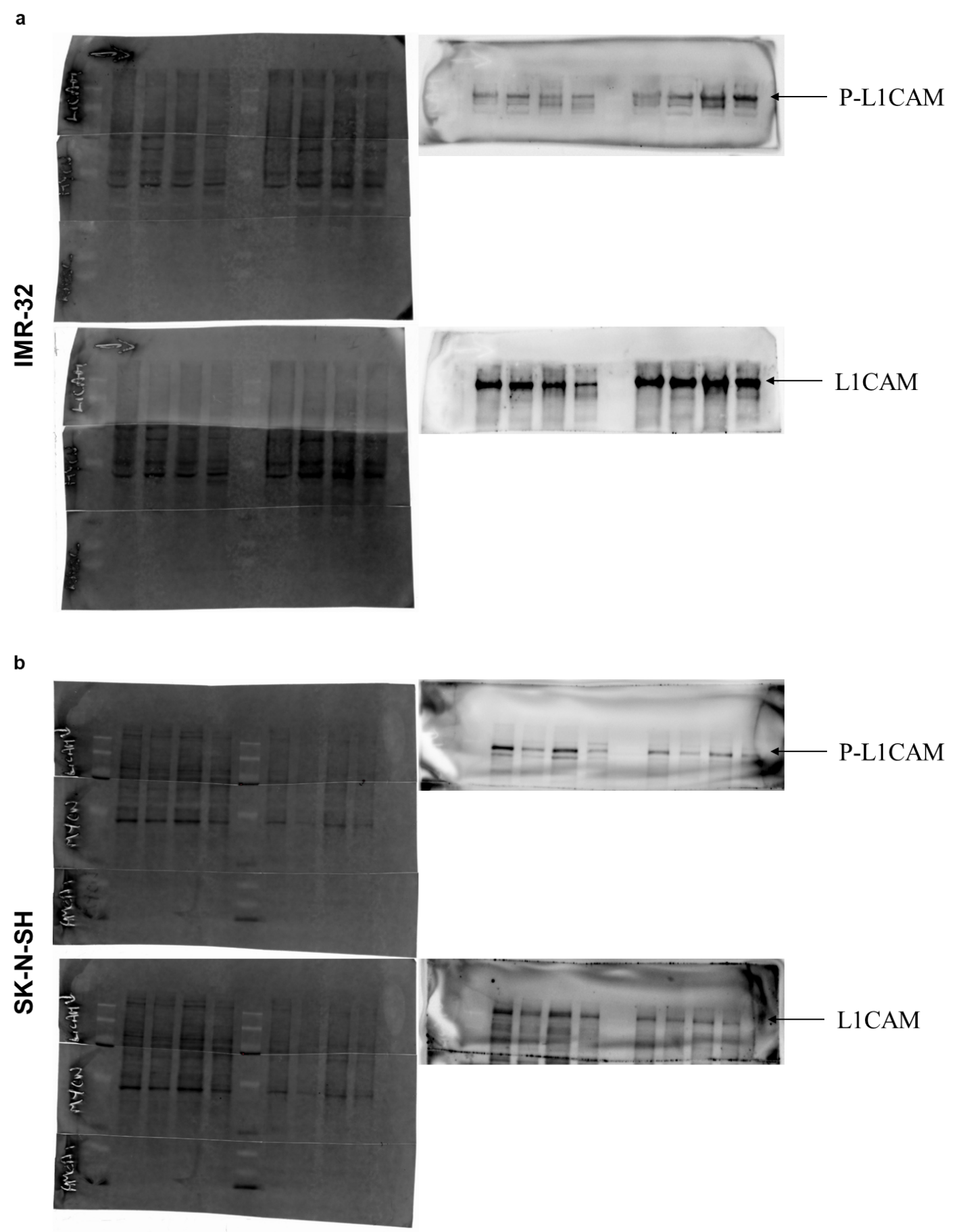


**Supplementary Figure 4.** Stain free blots and chemiluminescent images of p-L1CAM and L1CAM detected via western blotting in IMR-32 (a) and SK-N-SH (b) cells.


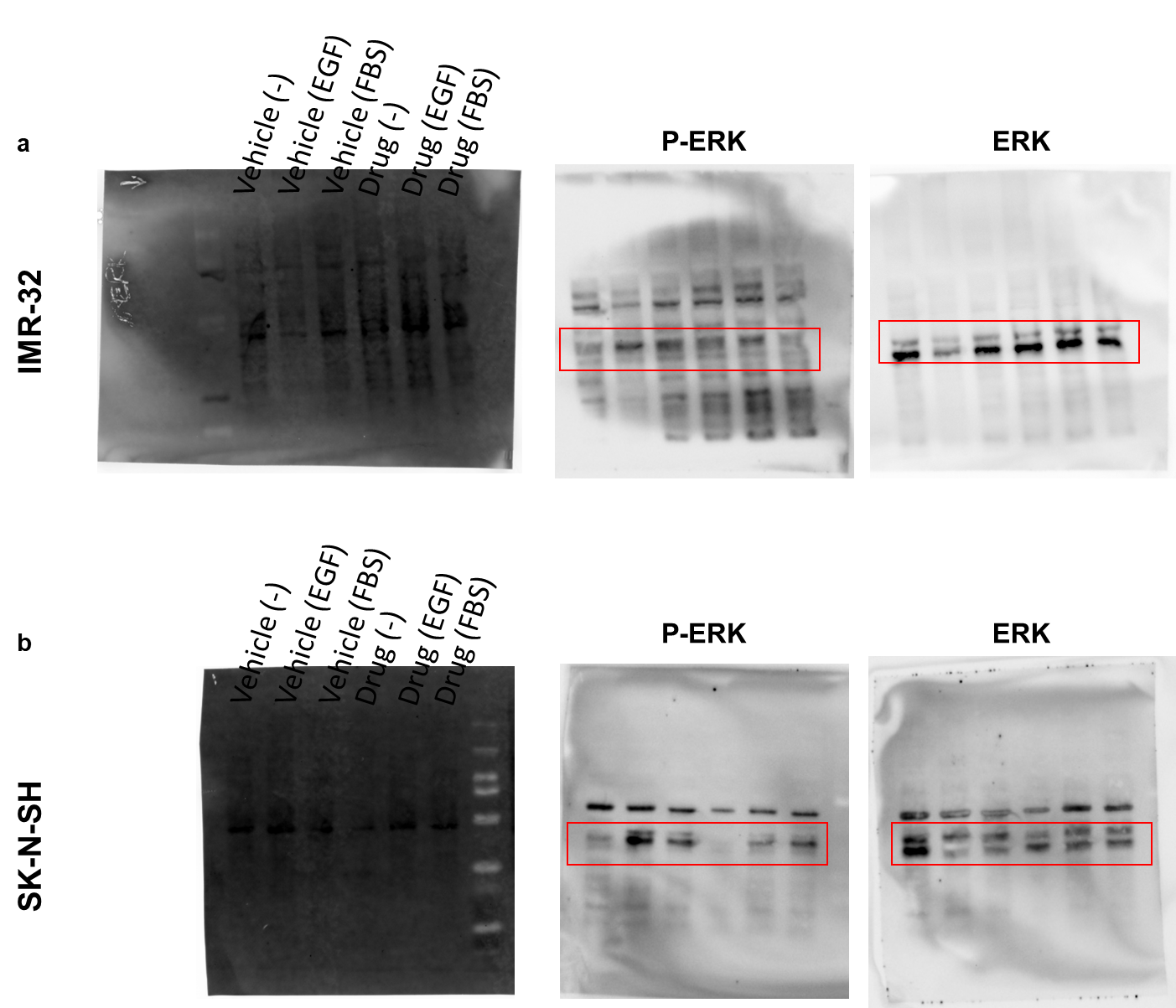


**Supplementary Figure 5.** Stain free blots and chemiluminescent images of p-ERK and ERK detected via western blotting in IMR-32 (a) and SK-N-SH cells (b).
